# The zebrafish mutants for the V-ATPase subunits d, ac45, E, H and c and their variable pigment dilution phenotype

**DOI:** 10.1186/1756-0500-6-39

**Published:** 2013-02-02

**Authors:** Jose L Ramos-Balderas, Samantha Carrillo-Rosas, Aida Guzman, Rosa E Navarro, Ernesto Maldonado

**Affiliations:** 1 Departamento de Biología Celular y del Desarrollo, Instituto de Fisiología Celular, Universidad Nacional Autónoma de México, Circuito Exterior, Ciudad Universitaria, México, D F 04510, Mexico

**Keywords:** Zebrafish, Insertional mutants, V-ATPase Subunits, Pigment dilution

## Abstract

**Background:**

The V-ATPase is a proton pump that creates an acidic medium, necessary for lysosome function and vesicular traffic. It is also essential for several developmental processes. Many enzymes, like the V-ATPase, are assemblies of multiple subunits, in which each one performs a specific function required to achieve full activity. In the zebrafish V-ATPase 15 different subunits form this multimeric complex and mutations in any of these subunits induce hypopigmentation or pigment dilution phenotype. We have previously found variability in the pigment dilution phenotype among five of the V-ATPase zebrafish mutants. This work presents additional information about such differences and is an update from a previous report.

**Findings:**

We describe the variable phenotype severity observed among zebrafish V-ATPase pigment dilution mutants studying mRNA expression levels from their corresponding genes. At the same time we carried out phylogenetic analysis for this genes.

**Conclusions:**

Based in the similarities between different pigment dilution mutants we suggest that there is an essential role for V-ATPases in melanosome biogenesis and melanocyte survival. Neither variable expression levels for the different V-ATPase subunits studied here or the presence of duplicated genes seems to account for the variable phenotype severity from this group of mutants. We believe there are some similarities between the pigment dilution phenotype from zebrafish V-ATPase insertional mutants and pigment mutants obtained in a chemical screening (“Tubingen pigmentation mutants”). As for some of these “Tubingen mutants” the mutated gene has not been found we suggest that mutations in V-ATPase genes may be inducing their defects.

## Background

Zebrafish pigment dilution mutants are the consequence of mutations in genes involved in vesicular traffic or endo-lysosomal function [1,2] and therefore they are tools to understand how melanosomes and other organelles are formed. This report is an update from data previously published by us, and others, about five V-ATPase mutants with a pigment dilution phenotype [3,4].

A large-scale retroviral insertional mutagenesis generated about 400 zebrafish mutants, which are currently at the “*Zebrafish International Resource Center*” and are used by several research groups to study different aspects of developmental biology and cell differentiation [5-8]. At the same time some of these insertional mutants are models for human diseases like oculocutaneous albinism [3], cancer [9,10], liver and kidney diseases [11,12] and neuroblastoma [13]. In total 16 insertional mutants have pigment defects and for 7 of these, insertions have been found tightly linked to subunits from the V-ATPase complex [8,14]. V-ATPase mutants have hypopigmentation or pigment dilution as the more visible phenotype [3] but also have retinal defects and some other physiological problems [4,15]. Since V-ATPase function has been found to be essential for melanosome biogenesis it was suggested that the pigment dilution phenotype is the consequence of a reduction in the number of melanosomes, which may affect melanocyte survival [3,4], the same is true for mutations in other genes also required for melanosome biogenesis [1,16]. Melanosomes are the organelles where melanin synthesis is carried out in the melanocyte (pigment cell) [17].

The V-ATPase complex is oriented in the membrane in such a way that it pumps protons from the cytoplasm out of the cell or to the interior of certain organelles. V-ATPase function is essential for secretion, lysosome function, vesicular traffic and phagocytosis [18]. V-ATPases are also important during development in invertebrates [19,20] and vertebrates [21]. Its function is required for specific processes like acquiring left-right asymmetry [22,23], biliary function [24], bone formation [25,26] and neural system development [27,28]. The vacuolar ATPase complex from vertebrates is composed of at least 15 subunits arranged in two functional domains: V1 with subunits A, B, C, D, E, F, G and H and V0 with subunits a, c, c”/b and e. Both domains have a combined molecular weight around 750 kDa. V-ATPases also have two accessory subunits known as the membrane proteins Ac45 and Ap2/M8-9. The V1 sector is peripheral to the membrane being the site for ATP hydrolysis and V0 domain is a multimeric membrane proton channel. V1 and V0 are connected by a stalk, which is formed by the V1 subunits C, E, G and H in the configuration CE_2_G_2_H. The assembly, interactions and function for each of the V-ATPase subunits have been reviewed extensively [18,29,30].

The seven known V-ATPase insertional mutants are: *atp6vod1*^*hi2188bTg*^ (V0-d1), *atp6ap1b*^*hi112Tg*^ (V0-ac45b), *atp6v1e1b*^*hi577aTg*^ (V1-E1b), *atp6v1h*^*hi923Tg*^ (V1-H), *atp6v0ca*^*hi1207Tg*^ (V0-ca), *atp6v1f*^*hi1988Tg*^ (V1-F) and *atp6ap2*^*hi3681Tg*^ (V0-ap2) (there are several alleles for each of these mutants, which are either insertional or chemically induced). Three more insertional mutants for V-ATPase subunits were recently obtained, which are: *atp6v1ba*^*la013065Tg*^ (V1-B), *atp6v1d*^*la013933Tg*^ (V1-D) and *atp6v0a1a*^*la015092Tg*^ (V0-a1a) [31]. Furthermore, knockdown morpholino experiments have also been carried out for V-ATPase subunits genes *atp6v0a1a*[28], *atp6v0b*[32], *atp6v0ca*[33], *atp6v0cb*[27] and *atp6v1a*[34]. This means that for 12 out of the 15 V-ATPase subunits there is a mutation or knock down experiment (the exceptions are V1-C, V1-G and V0-e subunits). Even though each mutant or morphant may have its own characteristics, pigment dilution is a common characteristic when V-ATPase function is affected.

In this work we present additional information about five of the V-ATPase insertional mutants and their genes. These are subunits V0-d1 (gene *atp6vod1*), V0-ac45b (gene *atp6ap1b*), V1-E1b (gene *atp6v1e1b*), V1-H (gene *atp6v1h*) and V0-ca (gene *atp6v0ca*).

## Materials and methods

### Fish husbandry and genotyping

All protocols carried out in animals were approved by the Office of Laboratory Animal Welfare (OLAW) from the National Institutes of Health (NIH) at the US, approval #A5281-01. Wild type zebrafish (strain TAB-14) and the five V-ATPase insertional mutant strains used in this work were obtained as a kind gift from Professor Nancy Hopkins of the Massachusetts Institute of Technology. Zebrafish embryos were obtained by natural crosses and then keep at 28.5°C, they were staged according to Kimmel et al. [35]. Adult zebrafish were maintained in a recirculation system (Aquatic Habitats) with constant pH, temperature and dark–light cycles [36]. Food consisted of harvested nauplii larvae mixed with macerated TetraminPro (Tetra). We genotyped fin clips to identify carriers by PCR as described before [1].

### Photography and dark adaptation assay

Live zebrafish larvae, WT and mutants, were placed in 3% methylcellulose (SIGMA) in excavated slides and oriented with a pin. No anesthetic was used because melanocyte appearance may be affected in the presence of tricaine. Photographs were taken in a StemiSV11 Zeiss Stereomicroscope using a Sony DSC-F707 camera attached to an adapter for microscopes (Martin Microscope Co.). Images were taken in the same light conditions for all larvae fish, photos were enhanced using Adobe Photoshop, however previous to any modification all images in the same figure were placed side by side in the same layer and only then enhanced together to avoid biased comparisons. The dark adaptation assay was carried out in 5 dpf larvae as described before [1]. In total 12 zebrafish larvae were used in this analysis, 1 WT and 5 mutants for each experiment, the assay was made in duplicate.

### RT-PCR and semi-quantitative RT-PCR

Total RNA was obtained using the Trizol method (Invitrogen), 60 WT zebrafish embryos were collected for each developmental stage, placed over ice for 20 min and then transferred directly to Trizol. For adult organ RNA extraction 12 adult fish were dissected, these were of 8 to 12 months old and both male and female were used indistinctly. Adult zebrafish were euthanized first in ice cold 0.025% in tricaine (3-aminobenzoic acid methyl ester from Sigma) as recommended [37]. TURBO DNase (Ambion) was added in the reaction mix to eliminate residual genomic DNA. Using 2 μg of total RNA we prepared first strand cDNA with oligo-dT primers and superscript III Reverse Transcriptase (Invitrogen). We always included a control without superscript III in order to confirm that prepared cDNA was free of genomic DNA as contaminant. In particular in brain and liver tissue from zebrafish it is difficult to eliminate genomic DNA and required double trizol extraction and repeated TURBO DNase treatments. Routinely RT-PCR was performed using a Taq enzyme and reagents from SIGMA. All experiments were repeated at least two times. The primers used to amplify actin were 5^′^- CATCAGCATGGCTTCTGCTCTGTATGG-3^′^ and 5^′^- GACTTGTCAGTGTACAGAGACACCCTG-3^′^. The primers used to amplify V1-d1 (gene *atp6vod1*) were 5^′^- GATTTGGATGAGATGAACATTGAGA-3^′^ and 5^′^- CACAATGTTCCGGCACTCTTGCTCC-3^′^. The primers used to amplify V0-ac45b (gene *atp6ap1b*) were 5^′^- GGAACCAGGCTGATCTAGCAAGCA-3^′^ and 5^′^-TCACGCTGAAGCTCTGGATCTGAA-3^′^. The primers used to amplify V1-E1b (gene *atp6v1e1b*) were 5^′^- CCAAACAGGGCGACTGGTATTTCA-3^′^ and 5^′^- GCCGACGTCCAGAAACAGATCAAG-3^′^. The primers used to amplify V1-H (gene *atp6v1h*) were 5^′^-TGTGGCTTCCAGCTGCAGTATCAG-3^′^ and 5^′^- ACTCCATTCCAGGCGTCCAGACTT-3^′^. The primers used to amplify the subunit V0-ca (gene *atp6v0ca*) were 5^′^-ACTCAGAGTCTAAACCTGCTAGACTG-3^′^ and 5^′^ GAGCCCCAGCACCTCTGCAAAGATC-3^′^. All RT-PCR products were in the range of 400 to 600 bp in size. An MJ-Research PTC-200 thermal cycler was used (25 cycles only for most reactions). RT-PCR was carried out with 2 μl of cDNA (25 μl final volume) but when semi-quantitative RT-PCR was performed the cDNA source was diluted 1:100 and 1:1000.

### Multiple alignments and phylogenetic tree building

Multiple alignments and Phylogenetic tree building were carried out using MacVector12. Protein sequences were used for multiple alignments and were obtained, by BLAST searches, from public databases (Ensembl and NCBI). Multiple alignments were made using ClustalW using the BLOSUM matrix with open gap penalty of 10%, extended gap penalty of 0.2% while the delay divergence was 30%. Phylogenetic trees were built with the Neighbor Joining method in the Best Tree mode with a systematic tie breaking.

## Findings

### Variable phenotype in the V-ATPase insertional mutants

**Figure 1:**
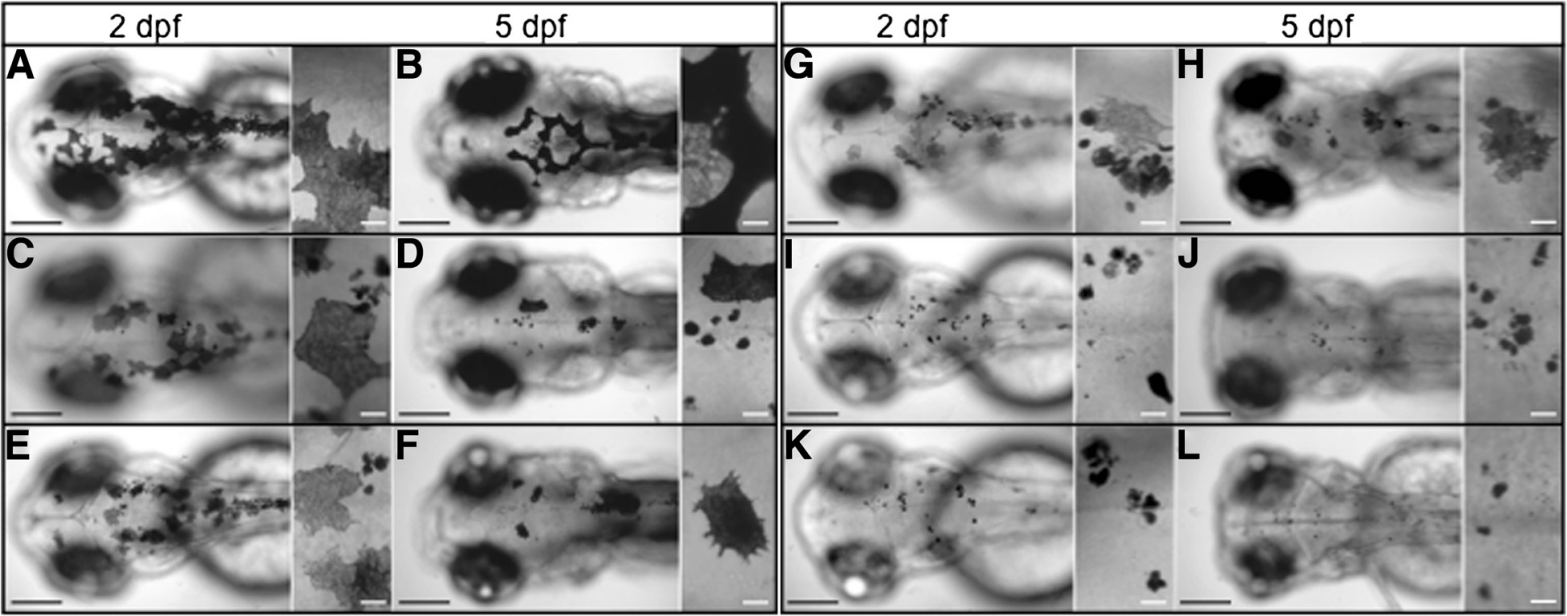
**Dorsal views from five insertional zebrafish mutants in V-ATPase subunits.** The fish larvae are shown at 2 dpf (**A**, **C**, **E**, **G**, **I** and **K**) and at 5 dpf (**B**, **D**, **F**, **H**, **J** and **L**). For each image a close up view is at the right side. Wild type larvae are in (**A** and **B**) and show normal pigmentation. Pigment dilution is observed in all V-ATPase mutants (**C** – **L**), however the severity of the pigment phenotype was mild in V0-d1 (gene *atp6vod1*) (**C** and **D**) and V0-ac45b (gene *atp6ap1b*) (**E** and **F**). It was more severe in V1-E1b (gene *atp6v1e1b*) (**G** and **H**) and V1-H (gene *atp6v1h*) (**I** and **J**), but even more severe in V0-ca (gene *atp6v0ca*) (**K** and **L**). In all the mutants there are a combination of pale melanocytes and dark melanin spots considered to be fragments of cells. Pale melanocytes became scarce the more severe the phenotype was (compare **C** and **I**). Bars in low amplification views are 200 μm and bars in close ups are 20 μm.

**Figure 2:**
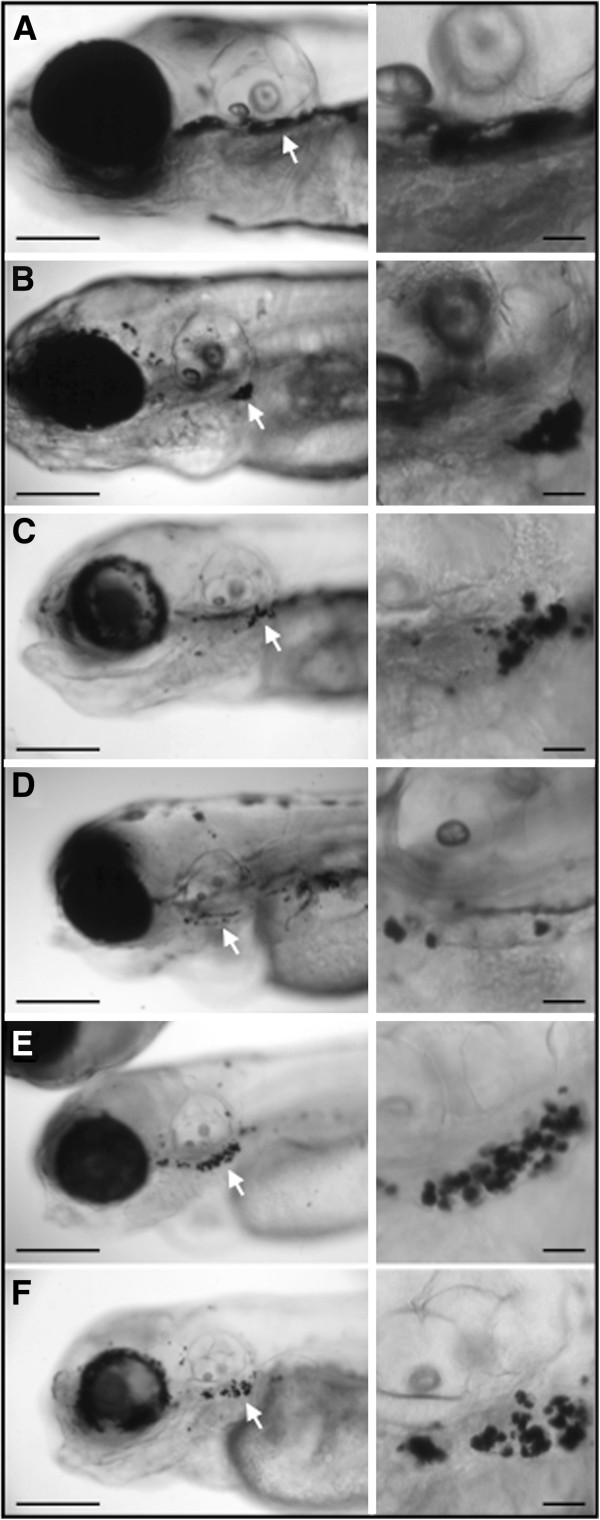
**Lateral views from zebrafish insertional mutants for five V-ATPase subunits.** Larvae WT **(A)** and mutants in subunits V0-d1 **(B)**, V0-ac45b **(C)**, V1-E1b **(D)**, V1-H **(E)** and V0-ca **(F)** are shown. To the right from every image there is a close up view of the region indicated by the white arrow in the left panel. Melanin round spots accumulate ventrolateral to the ear, which is an identical feature observed in the class VI.C of zebrafish mutants from the Tubingen chemical screening (see reference 38). Even though the reason for this phenotypic characteristic is unknown, it could be used as a form to identify this type of mutations.

The zebrafish V-ATPase insertional mutant were found during a retrovirus mutagenic screening, they show pigment dilution, developmental delay and retinal degeneration [1,3,4]. We present additional information for five of these mutants in subunits V0-d1, V0-ac45b, V1-E1b, V1-H and V0-ca from the V-ATPase complex. Pigment dilution could be observed as early as 15–18 hpf, in these mutants, before that, they are undistinguishable from WT embryos. We detected that not all V-ATPase subunits mutants have the exact same phenotype. The mutants in V1-H and V0-ca subunits have a reduced number of melanocytes and those still remaining are pale in appearance, at the same time, these mutants have small irregular dark melanin spots (Figure 1I – L, see close ups). While the mutants for V0-d1, V0-ac45b and V1-E1b have normal melanocytes in early developmental stages that later became pale and fragmented (Figure 1C – H), the V1-H and V0-ca mutants always show reduction in melanocytes and conspicuous melanin spots (Figure 1I and 1K). These melanin spots that are 3 to 15 μm in size, which were reported previously in some zebrafish mutants (class VI.C) from the Tubingen chemical screening, where it was suggested to be the consequence of melanocyte progressive degeneration [38]. In any case, some V-ATPase insertional mutants showed a more severe pigment dilution phenotype than others and we decide to study this event. It is worth mentioning that it has been reported that these five V-ATPase mutants have either null mRNA expression (V1-E1b, V1-H and V0-ca) or the produced mRNA is not functional (V0-d1 and V0-ac45b) [4]. As we mention before the V1-H mutant in the “severe pigment dilution” group, it is interesting that this particular subunit was reported to be not essential for the assembly of the V-ATPase complex [39,40]. We also observed that in our set of V-ATPase mutants, some melanin spots usually collect at the ear ventrolateral region (Figure 2), although we do not have an explanation for this, it is interesting that such characteristic was also reported for all zebrafish pigment mutants from the class VI.C Tubingen screening [38].

Variability in the phenotype severity could also be observed in developmental delay, because the mutants for V1-H and V0-ca subunits, have the shortest body size and were more affected in general development (see Additional file 1: Figure S1). As a further test for variability in phenotype severity we performed a dark adaptation assay in the V-ATPase mutants. When larvae are exposed to a dark environment melanosomes become broadly distributed and the melanocytes look expanded, this is consequence of a physiological hormonal response that involves the retina and the pituitary gland [41]. We observed that zebrafish V0-d1, V0-ac45b mutants expand their melanocytes but V1-E1b, V1-H and V0-ca mutants did not present a comparable response (see Additional file 2: Figure S2). It is worth mentioning that while carrying out this experiment we notice that, in all the mutants, melanin spots remain unresponsive to the dark adaptation assay, supporting the idea that melanin spots are the consequence of melanocyte degeneration.

### mRNA expression for the V0-d1, V0-ac45b, V1-E1b, V1-H and V0-ca V-ATPase subunits

**Figure 3:**
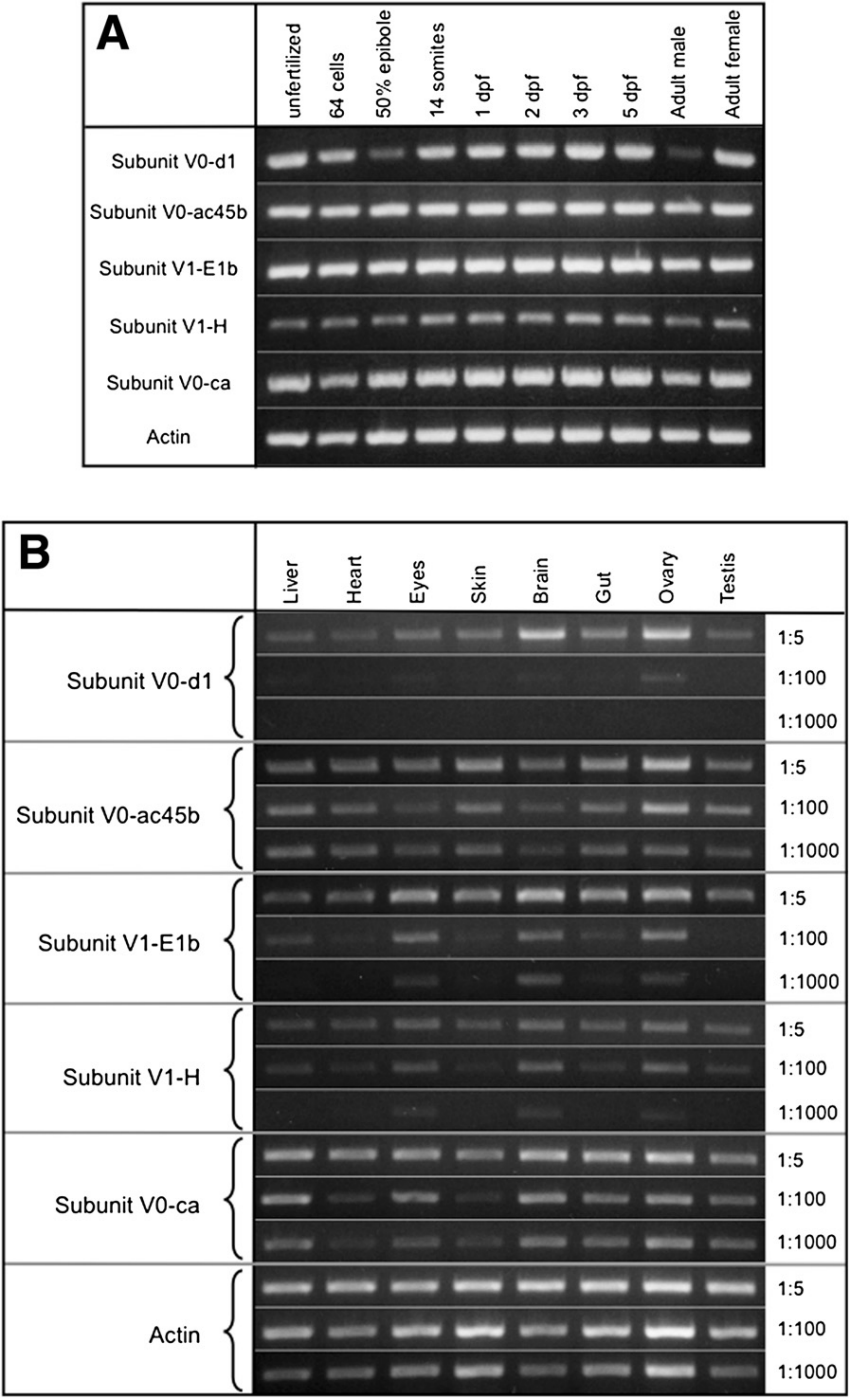
**RT-PCR of the five V-ATPase subunits in embryos at different developmental stages and in organs from adult fish. (A)** RT-PCR from WT embryos at different developmental stages using oligonucleotides that amplify five different V-ATPase subunits. All subunits are consistently expressed throughout all developmental stages, however subunit V1-H is expressed in lower amounts than V0-d1, V0-ac45b, V1-E1b, V1-H and V0-ca. Alpha actin expression was used as an expression control. V0-d1 is abundant in adult males than in females. **(B)** Semi-quantitative RT-PCR from total RNA obtained from liver, heart, eyes, skin, brain, gut, ovaries and testis from adult zebrafish. The cDNA used was diluted 1:5, 1:100 and 1:1000 and then used for the RT-PCR. V-ATPase subunits V0-ca and V0-ac45b have the higher expression levels, in contrast V0-d1 have the lower expression levels. V1-E1b and V1-H have similar expression; both are enriched in the eyes, brain and ovary.

We decide to explore the possibility that different WT expression levels in the V-ATPase V0-d1, V0-ac45b, V1-E1b, V1-H and V0-ca subunits may explain phenotypic differences in their respective mutants, therefore we decided to look at the expression for these five genes at the mRNA level in WT zebrafish embryos from different developmental stages and as well from different zebrafish adult organs. First we found that the five mRNAs are maternally provided because unfertilized eggs show positive expression by RT-PCR (Figure 3A). All the V-ATPase subunits studied here were steadily expressed during the initial five days of development except for V0-d1 subunit that has a reduced expression at 50% epiboly, which may indicate expression changes during the midblastula transition. All subunits showed high expression levels during development except for V1-H, which seems to be less expressed (Figure 3A). We performed semi-quantitative RT-PCR in samples of total mRNA obtained from liver, heart, eyes, skin, brain, gut and gonads from WT adult zebrafish. Different dilutions of cDNA (1:5, 1:100 and 1:1000) were used as a source for PCR with the idea that persistent bands in the samples with less cDNA represent higher levels of expression. We observed that V0-ac45b and V0-ca have the highest expression levels, in most organs, of all the subunits tested (Figure 3B), however V0-ac45b belongs to the less severe phenotype subgroup and V0-ca to the more severe phenotype subgroup. The lowest expression observed was for the V0-d1 subunit (low amplification at 1:100 and 1:1000 dilutions) with some increased levels in brain and ovary. V1-E1b and V1-H have similar expression patterns, being expressed in higher levels in the eyes, brain and ovary (Figure 3B). We did not find any correlation between mRNA expression levels and phenotypic variability, however several other explanations are possible. Some proteins may have multiple functions, which also could explain phenotype variation. It has been shown that V0-c subunits have an additional function in the cell, which is independent of being part of the V-ATPase complex. V0-c proteins induce, by themselves, membrane fusion events [42,43], therefore high expression levels must be expected for V0-c subunits and when mutated, more cell functions could be affected and not just V-ATPase inhibition. This may be also true for other V-ATPase subunits like V0-a1, which was reported to mediate fusion between phagosomes and lysosomes in zebrafish and such activity did not depend of proton pumping or ATP consumption [28].

### Gene duplications in some V-ATPase subunits

Sometimes having duplicated genes with redundant functions may ameliorate a phenotype when one of the alleles gets a mutation, as the nodal zebrafish genes *cyclops* and *squint*, in which only the double mutant shows a similar phenotype to the mouse nodal mutant. Mice have a single nodal copy while zebrafish possess two [44]. We search the zebrafish genome (*sanger center, version Zv9*) looking for duplicated genes for the five V-ATPase subunits studied here. We found that V0-ac45b, V1-E1b and V0-ca have duplicated genes (V0-ac45a, V1-E1a and V0-cb respectively) while V0-d1 and V1-H only were present in a single copy (Table 1). We did not find any clear correlation between the variable severity in the V-ATPase phenotypes (pigment-dilution and developmental-delay) and the number of duplicated genes (Table 1) however, It is possible that V0-ac45 subunits “a” and “b” could have redundant protein functions, hence the less severe phenotype, while subunits V1-E and V0-c may not have any redundancy and show more severe phenotypes, specially V0-ca mutant. To test this hypothesis we will have to study not only protein expression but also the V-ATPase function with different compositions of the oligomeric complex.

**Table 1:** Zebrafish mutants used in this study, their phenotype severity and duplicated genes

**Mutant name**	**Gene name**	**Protein name**	**Chromosome position**	**Phenotype severity**	**Duplicated gene**	**Protein name**	**Chromosome position**	
*atp6vod1*^*hi2188bTg*^	*atp6v0d1*	V0-d1	7:36.5	*	-	-	-	
*atp6ap1b*^*hi112Tg*^	*atp6v0ap1b*	V0-ac45b	23:26.3	*	*atp6v0ap1a*	V0-ac45a	23:4.9	
*atp6v1e1b*^*hi577aTg*^	*atp6v1e1b*	V1-E1b	4:5.1	**	*atp6v1e1a*	V1-E1a	25:16.9	
*atp6v1h*^*hi923Tg*^	*atp6v1h*	V1-H	2:30.5	**	-	-	-	
*atp6v0ca*^*hi1207Tg*^	*atp6v0ca*	V0-ca	3:15.6	***	*atp6v0cb*	V0-cb	24:40.7	

*less severe.

**mildly severe.

***strongly severe.

**Figure 4:**
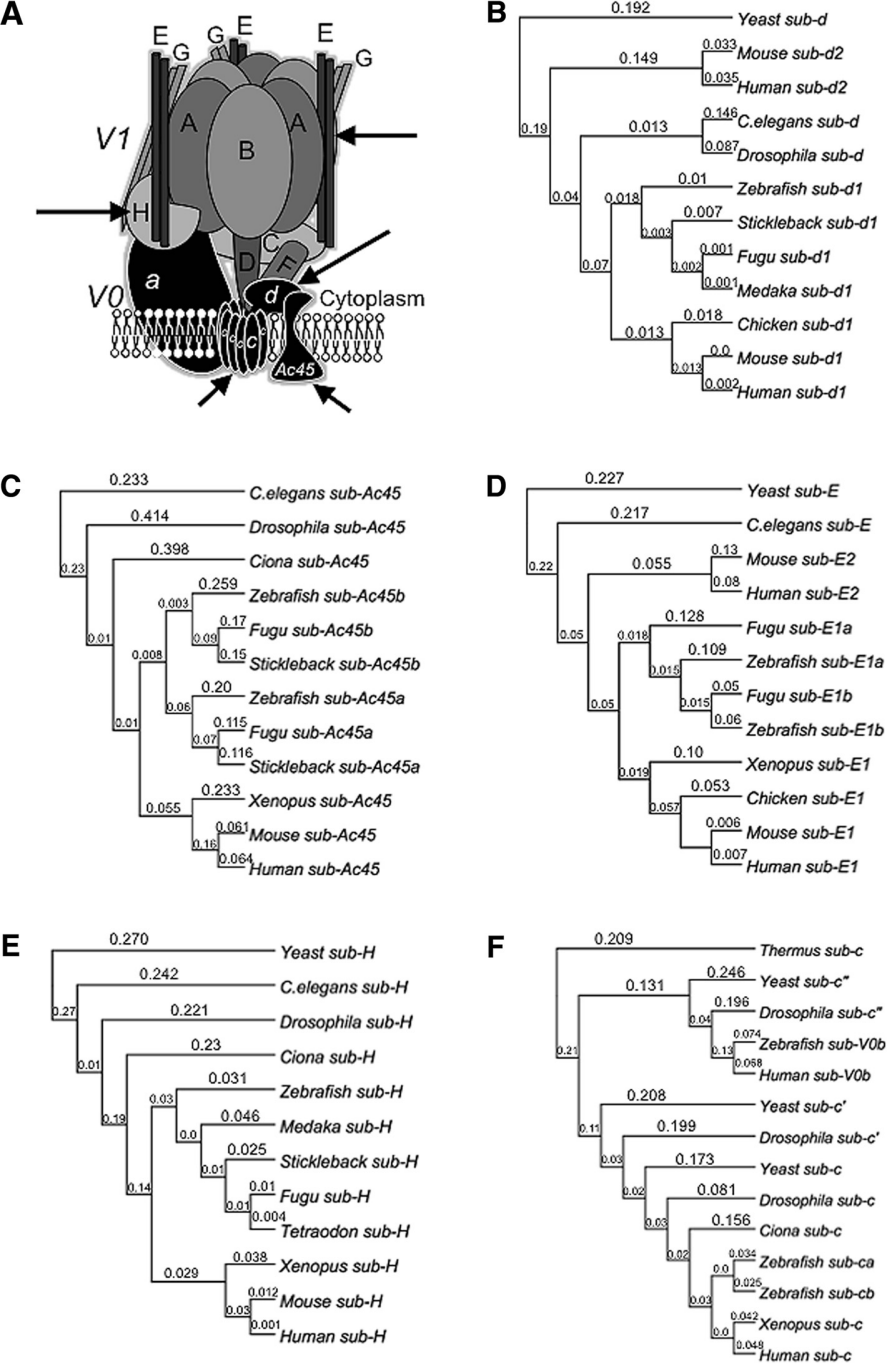
**Phylogenetic analysis of five V-ATPase subunits genes. (A)** Schematic representation showing the proposed subunit arrangement in the V-ATPase complex. Phylogenetic trees were made using the aminoacid sequence from the five V-ATPase subunits from different organisms. The analysis was made using MacVector 12.5, multiple sequence alignment was carried out with ClustalW and trees were built with the Neighbor Joining method using a Best tree with systematic tie breaking. Subunits V0-d1 **(B)** and V1-H **(E)** have a single copy in the zebrafish genome while subunits V0-ac45b **(C)**, V1-E1b **(D)** and V0-ca **(F)** had duplication events and possess two different copies. Aminoacid sequences were obtained from Ensembl or NCBI databases. The branch labels are substitutions per site. Outer groups used for subunits V0-d1, V1-E1b and V1-H were Yeast, for V0-ac45b *C. elegans* and for V0-ca the *Thermus thermophilus* bacteria.

In order to further analyze V-ATPase subunits gene duplications we build phylogenetic trees. Subunit V0-d1 is duplicated in mammals (V0-d1 and V0-d2) (Figure 4B) as in plants and *Drosophila* (not shown) but not in teleost fishes (like zebrafish) [45], although there is only one copy of V0-d subunit in the zebrafish genome and could be named V0-d we choose to keep the V0-d1 name because the zebrafish gene belongs to the d1 cluster and not to the d2 cluster (Figure 4B). Subunit V0-ac45b is duplicated in teleost fish but not in pre-chordates (*Ciona intestinalis*) nor in mammals. It was reported that V0-ac45 has a specific function in bone reabsorption [25] and in POMC processing and secretion [46], because we found the V0-ac45b is highly expressed in ovary and skin (Figure 3B) it would be interesting to determine if V0-ac4b has any other specific functions in these organs. The V-ATPase subunit V1-E is duplicated in teleost fishes, however our phylogenetic analysis shows that this is a different duplication event that the one from mammals (Figure 4D). While the mammal’s gene duplication produced V1-E1 and V1-E2, both zebrafish copies are located in the V1-E1 group, suggesting that the zebrafish genes originated from a duplication of V1-E1 ancestor or that V1-E2 genes have diverged more rapidly than V1-E1 genes. In any case the zebrafish genes duplicates must be named V1-E1a and V1-E1b (and not V1-E1 and V1-E2). The V1-E subunit mutant described in this work corresponds to the V1-E1b gene. Subunit V1-H is highly conserved and did not have any duplicated gene in zebrafish or in any other organisms we look (Figure 4E). There are three V0-c subunit genes in Yeast, *Drosophila melanogaster and Caenorhabditis elegans* (known as V0-c, V0-c’ and V0-c”). There is no V0-c’ copy in vertebrates and the V0-c” subunit, in humans, is known as V0-b because inverted commas could not be used in human gene names according to nomenclature rules [47]. We find that zebrafish only has a single copy of subunit V0-c” but duplicated genes for V0-c, which we refer as V0-ca and V0-cb (Figure 4F), the insertional mutation studied here corresponds to the V0-ca gene. All other teleost fishes (Fugu, Stickleback, Medaka, etc.) have the same number of V0-c V-ATPase subunits as in zebrafish (not shown). It was reported that the duplicated gene V0-cb is preferentially expressed in the zebrafish brain, being more specific at the presynaptic vesicles and is regulated by Notch signaling [27]. According to our RT-PCR data V0-ca has a ubiquitous expression (Figure 3B).

## Conclusions

We described here that there is a variable severity in pigment dilution and developmental delay between the five V-ATPase mutants even though these mutants do not possess functional mRNA for the affected genes [4]. Variability in phenotype severity suggest that V-ATPase function is not equally impaired in all these mutants and that each subunit may play its functional role only in some developmental stages, only in some organs or only in some physiological conditions. It also reveals how plastic is the functionality of the V-ATPase and how complex is regulation could be.

We find similarities between the zebrafish five V-ATPase insertional mutants and the Tubingen VI.C mutants (previously published by others); both groups of mutants show melanin spots that collect ventrolaterally to the ear (Figure 2), in the hindgut and in clusters in the dorsal stripe (not shown). Mutants in the class VI.C that were originally found in the Tubingen screening are *delayed fade* (*dfd*), *fade out* (*fad*), *fading vision* (*fdv*) and *Quasimodo* (*qam*) [38]. Only the *fdv* mutation has been positionally cloned and it was found to be the consequence of a premature stop codon halfway the *silva/pmela* gene, which codes for a protein required for intraluminal fibril formation required for melanosome maturation [16]. Due to the similarities between the V-ATPase mutants and the Tubingen class VI.C mutants we believe some of the yet uncloned pigment dilution mutants (*dfd*, *fad* or *qam*) may harbor mutations in V-ATPase subunits. The *fad* mutant shows retinal degeneration [48], which could also be observed in some of the insertional V-ATPase mutants [15]. It was reported that the chemically induced zebrafish mutant *a82* has a pigment dilution phenotype and therefore was a candidate for mutations in a V-ATPase subunit, so it was tested by complementation crosses against several V-ATPase mutants and found to be an allele of V0-ac45b [4].

V-ATPase function must be essential for melanosome biogenesis, the lack of such organelles cause the pigment dilution phenotype and may also induce melanocyte degradation, which is observed in the form of melanin spots in the zebrafish pigment dilution mutants [1,2,38]. It has been shown that pre-melanosomes are more acidic than mature melanosomes, which suggests they could have high requirements for V-ATPase activity, furthermore the V-ATPase subunit V0-a3 has been physically found in melanosomes from mouse melanocytes [49].

We also described that the variable pigment dilution phenotype, in this mutant collection, could not be explained by differences in mRNA expression of the analyzed V-ATPase subunits and although some of these subunits have duplicated genes, it does not seem to exist any functional redundancy that lessen the phenotypic severity observed, as it has been shown for other genes [44]. With the sole exception of V0-a45b, in which, the phenotype is among the less severe and has a duplicated V0-a45a that may be compensating for the lack of V0-a45b, however this will need confirmation. It is common that zebrafish mutants with specific phenotypes are induced by mutations that affect the expression of transcription factors, ligands or receptors [50]. This collection of pigment dilution zebrafish mutants shows that there is a new set of models with a very specific phenotype named “pigment dilution” induced when melanosome biogenesis is affected, and as a group could be used to get a better understanding about melanosome biogenesis and how multimeric enzymes achieve staggering levels of functional plasticity.

## Abbreviations

V-ATPase: Vacuolar-ATPase enzyme; ATP: Adenosin Triphosphate; TAB-14: Tubingen-AB-14 zebrafish strain; kDa: Kilodaltons; bp: Base pairs; cDNA: Complementary DNA; WT: Wild type; PCR: Polymerase chain reaction; Hpf: Hours post-fertilization; Dfp: Days post-fertilization; ENU: Ethyl-nitrosourea; POMC: Propiomelanocortin; V1-A, V1-B, V1-C, V1-D, V1-E1b, V1-F, V1-G, V1-H: Subunits A B, C, D, E1b, F, G H from the V1 domain of the V-ATPase; V0-a1a, V0-ca, V0-cb, V0-c”/b, V0-d1, V0-e: Subunits a1a ca, cb, c”/b, d1 and e from the V0 domain of the V-ATPase.

## Competing interests

The authors declare that they do not have competing interests.

## Authors’ contributions

EM and REN conceived, designed the experiments and wrote the manuscript. REN and AG carry out the fish larvae photography, EM did the dark adaptation experiment, JLRB carry out the RT-PCR experiments, EM and SCR did the phylogenetic analysis and all authors read and approved the final manuscript.

## Supplementary Material

Additional file 1: Figure S1 Developmental delay and body size reduction in V-ATPase mutants. Dorsal views of the whole body from zebrafish WT (A) and five different V-ATPase mutants (B – F) at 5 dpf. A consequence of developmental delay is that by 5 dpf the mutant larvae have not reached is full size. Zebrafish mutants for subunits V0-d1 and V0-ac45 were of the same size as WT fish (A – B), while V1-E1b and V1-H mutants have a slight reduction in body size (D and E). V0-ca mutant is the most affected of all and its size is much more reduced. Bar is 400 μm. Click here for file

Additional file 2: Figure S2 Dark adaptation experiment in V-ATPase zebrafish mutants. Background adaptation was used as a qualitative assay to compare the physiological state of V-ATPase mutants at 5 dpf. (A) WT, (B) V0-d1, (C) V0-ac45b, (D) V1-E1b, (E) V1-H and (F) V0-ca. Larvae at the right panels are the same fish than the left panels, after 2 h of dark adaptation. Whereas WT, V1-d and V0-ac45b larvae (A – C) show a positive response by expanding melanocytes in the dark background, V1-E1b, V1-H and V0-ca fail to show any response. White arrows point at the same melanocyte cell before and after dark adaptation. In mutant fish there are as well spots of melanin that we consider fragments of cells (black arrows) these spots do not expand after the dark adaptation treatment. Because of the pigment dilution phenotype in V-ATPase mutants, melanocytes are paler than WT melanocytes. Images from before and after dark adaptation were taken under the same light conditions. Bar is 200 μm. Click here for file
