# Supplementary figures and images for: The zebrafish mutants for the V-ATPase subunits d, ac45, E, H and c and their variable pigment dilution phenotype
Source: BMC Res Notes. 2013 Feb 2;6:39. doi: 10.1186/1756-0500-6-39 (PMC3599454; doi:10.1186/1756-0500-6-39)

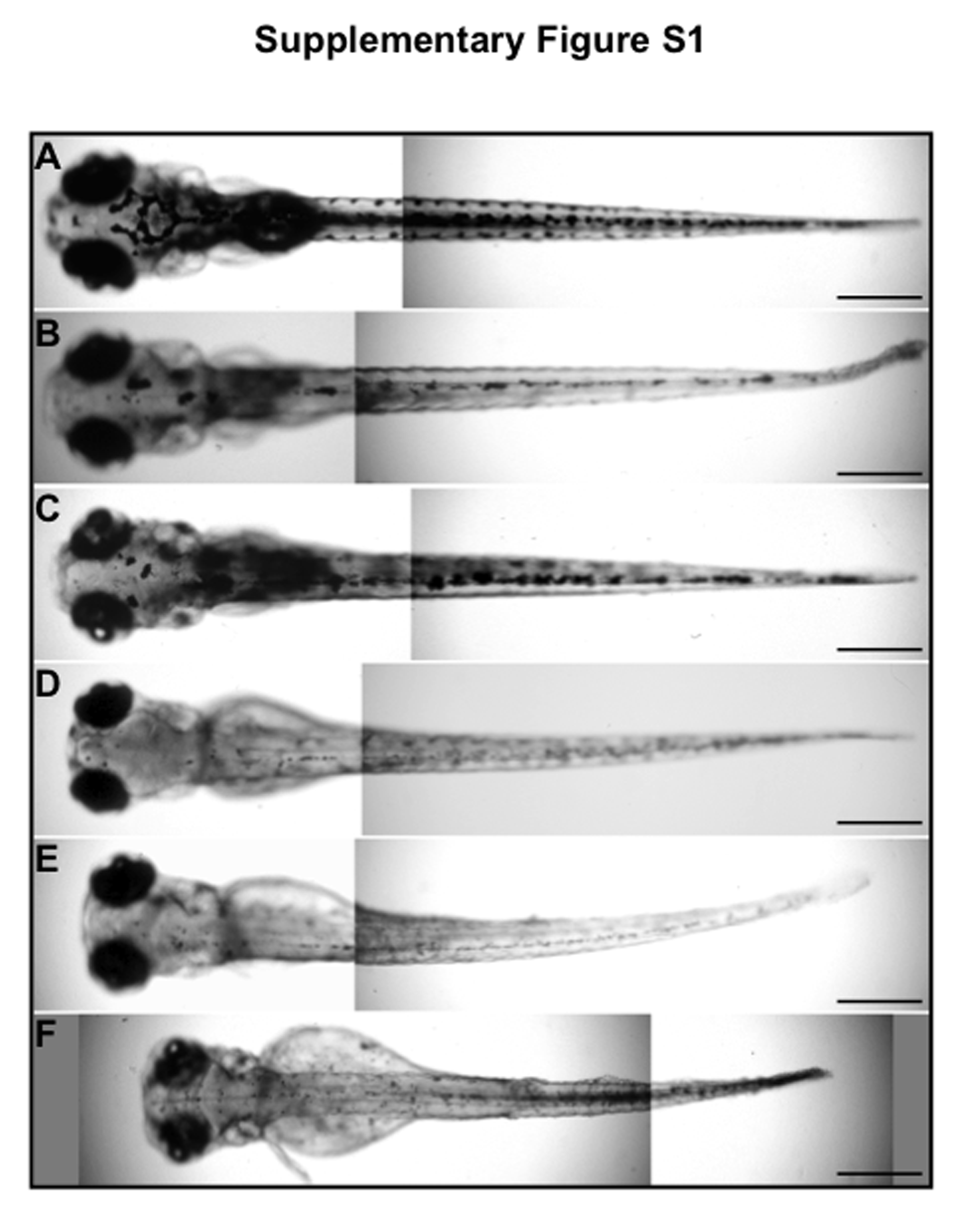

Supplement: Additional file 1: Figure S1 — Developmental delay and body size reduction in V-ATPase mutants. Dorsal views of the whole body from zebrafish WT (A) and five different V-ATPase mutants (B – F) at 5 dpf. A consequence of developmental delay is that by 5 dpf the mutant larvae have not reached is full size. Zebrafish mutants for subunits V0-d1 and V0-ac45 were of the same size as WT fish (A – B), while V1-E1b and V1-H mutants have a slight reduction in body size (D and E). V0-ca mutant is the most affected of all and its size is much more reduced. Bar is 400 μm. [file 1756-0500-6-39-S1.tiff]

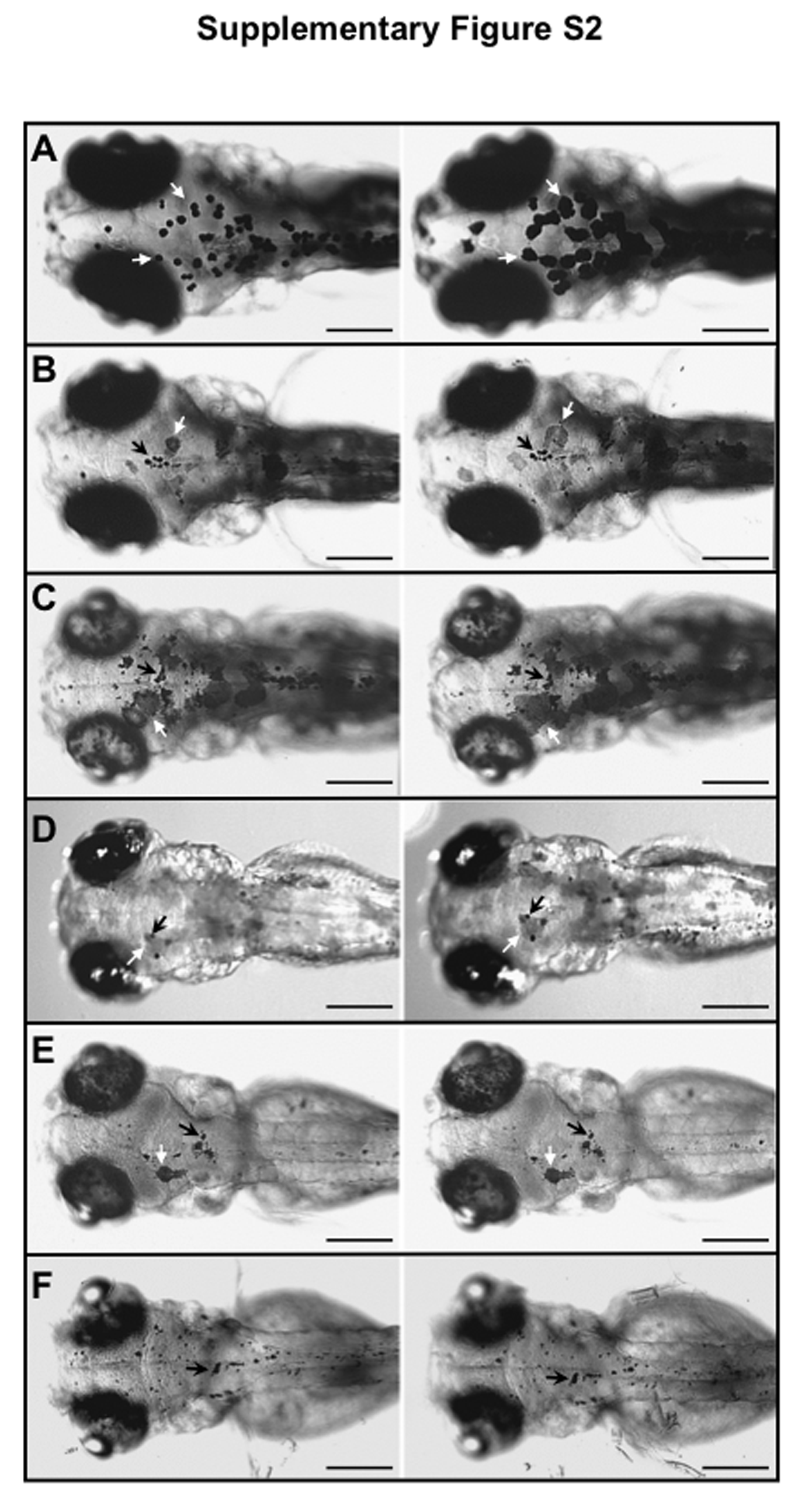

Supplement: Additional file 2: Figure S2 — Dark adaptation experiment in V-ATPase zebrafish mutants. Background adaptation was used as a qualitative assay to compare the physiological state of V-ATPase mutants at 5 dpf. (A) WT, (B) V0-d1, (C) V0-ac45b, (D) V1-E1b, (E) V1-H and (F) V0-ca. Larvae at the right panels are the same fish than the left panels, after 2 h of dark adaptation. Whereas WT, V1-d and V0-ac45b larvae (A – C) show a positive response by expanding melanocytes in the dark background, V1-E1b, V1-H and V0-ca fail to show any response. White arrows point at the same melanocyte cell before and after dark adaptation. In mutant fish there are as well spots of melanin that we consider fragments of cells (black arrows) these spots do not expand after the dark adaptation treatment. Because of the pigment dilution phenotype in V-ATPase mutants, melanocytes are paler than WT melanocytes. Images from before and after dark adaptation were taken under the same light conditions. Bar is 200 μm. [file 1756-0500-6-39-S2.tiff]
